# Effectiveness of the Positive deviance and parent facilitator training strategies on the nutritional status of children and youth with cerebral palsy: A quasi-randomised trial with a factorial design

**DOI:** 10.1371/journal.pgph.0005027

**Published:** 2025-08-19

**Authors:** Lukia Hamid Namaganda, John Ssenkusu, Asige Elizabeth, Carin Andrews, Angelina Kakooza Mwesige, Fred Wabwire Mangen, Hans Forssberg

**Affiliations:** 1 CURIE Study Consortium, Iganga-Mayuge Health and Demographic Surveillance System, Iganga, Makerere University, Uganda; 2 Department of Epidemiology and Biostatistics, Makerere University, Kampala, Uganda; 3 Department of Pediatrics and Child Health, Makerere University, Kampala, Uganda; 4 Department of Women’s and Children’s Health, Karolinska Institutet, Stockholm, Sweden; 5 Department of Women’s and Children’s Health, Uppsala University, Sweden; PLOS: Public Library of Science, UNITED STATES OF AMERICA

## Abstract

High malnutrition among children with CP in low-income countries underscores the need for community-based nutrition strategies. This study aimed to describe the effectiveness of two caregiver-led interventions including, the positive deviance (PD) and the parent facilitator training (PFT) interventions on malnutrition among children and youth (C&Y) with CP in rural Eastern Uganda. This was a 2x2 factorial quasi-randomized trial among 124 pairs of caregiver-malnourished C&Y with CP aged 2–24 years, at the Iganga Mayuge Health and Demographic Surveillance Site (IMHDSS) in Eastern Uganda. Outcome measures included three months change in weight gain, Weight for-age-z scores and Body Mass Index for-age-z scores. Change in weight status was modelled in a multiple linear regression adjusting for baseline characteristics. Non-factorial analysis was used to determine the effect of combining both interventions on weight status. The interaction effect between PD and PFT was not statistically significant (P > 0.05). Those who received the PD intervention significantly gained more weight by 520g (adjusted coefficient = 0.52, 95%CI 0.16,0.88, p = 0.005) and improved their BMI for age z-scores (adjusted coefficient = 0.65, 95% CI 0.35,0.94, P < 0.01) than those who did not, while those who received PFT significantly improved their Weight for Age z-scores (adjusted coefficient = 0.42,95%CI 0.14,0.56, p = 0.006). Non-factorial analysis revealed a significant higher weight gain (770g) and improved BMI for age z-scores among those who received both interventions (adjusted coefficient = 0.77, 95%CI 0.22.1.37, p = 0.009). The PD alone or combined with PFT interventions improves weight status better than the PFT intervention alone. The PD and PFT caregiver-led strategies should be merged into existing community-based nutrition programs to reduce the high burden of malnutrition among C&Y with CP in low-income settings.

## Introduction

Cerebral palsy (CP) is a common neurodevelopmental disorder with an estimated prevalence of 3.1-3.7 per 1000 children in sub-Saharan Africa and other low- and middle-income countries [1, 2]. In addition to core motor impairments, and other associated impairments, children with CP face risks of malnourishment and retarded growth [3,4]. In a recent report from Uganda, approximately two thirds of children with CP were malnourished and exhibited a slower growth, especially those with severe motor impairments and feeding difficulties needing assistance for feeding [3], in accordance with earlier studies [5–7]. Previous studies have indicated additional manageable causes of malnutrition including inadequate food and dietary intake that alter energy metabolism [8–10]. Poor management of malnutrition among children with CP diminish their ability to thrive and survive [11–14], thus interventions are imperative.

Global efforts towards childhood malnutrition have been successful but not disability-inclusive [15]. The United Nations Sustainable Development Goals (SDGs) strongly address global health inequality [16], but gaps still exist between policy and implementation of nutrition programs for individuals with disabilities. Exclusion of children with disabilities in guidelines partly depends on lacking information on malnutrition and how to manage malnutrition in this population [15]. A recent systematic review found only eight articles relating to nutrition interventions among children with CP in low-and middle-income countries, yet none of these was from low-income countries due to scarcity in documentation [17]. Children with CP require targeted interventions for both their nutrition and disability specific needs, thus targeting nutrition alone has not been effective in this population [18]. Interventions related to feeding elicit better nutritional outcomes among children with CP [19–22], but majority are facility based, and poorly accessed by caregivers in low income settings [23]. This underscores the need for community-based approaches.

Malnutrition is related to severe motor impairments, [10,24] yet it is not clear whether interventions improving motor functions promote better nutritional outcomes. Locally derived nutrition specific interventions like the positive deviance (PD) strategy indicate weight gain of typically developed children [25–27], but effectiveness among children with CP is still unclear. The PD strategy operates on the premise that solutions to nutritional challenges in any community lie within families of well-nourished children residing in the same community. Such families portray nutritional resilience, while using adaptable, culturally acceptable and sustainable solutions [28]. Several studies have reported improved nutritional status and weight gain of>200g, or>900g after 12 days or 3 months, respectively, following the PD nutrition intervention largely among malnourished typically developed children aged 6–36 months [25, 26]. Our study tested the effectiveness of two caregiver-led interventions on the nutritional status of C&Y with CP in Eastern Uganda, i.e., the positive deviance nutrition strategy (PD), and the parent facilitator training (PFT) of the Akwenda Intervention Program [29]. We specifically aimed to 1) Determine the effect of PD and PFT, respectively, on the weight status of C&Y with CP after three months, 2) Determine whether combining the PD and PFT strategies had additional effect on the weight status of C&Y with CP after three months.

## Methods

### Ethics Statement

Ethical approval for this study was obtained from Makerere University School of Public Health Higher degrees and Research Ethical Committee (HDREC 186), and the Uganda National Council for Science and Technology (protocol REF: HS1427ES). The trial has been registered with Clinical Trials.gov at NCT05592834. This trial was conducted between August 2022 to October 2022 after which outcome evaluations were conducted. All the foods used during the PD intervention were locally produced, and well tolerated by C&Y with no harmful effects attributed to these foods during the study period. Caregivers were encouraged to practice what they had learned at home during non-group session days each week.

### Study design and setting

This was a 2x2 factorial quasi-randomised trial assessing the nutritional effectiveness of the PD and PFT strategies within four experimental conditions including, i) Both PD and PFT, ii) Only PD, iii) Only PFT, and iv), Neither PD nor PFT (controls). This trial was conducted within the districts of Iganga, Mayuge and Bugweri that make up the Iganga Mayuge Health and Demographic Surveillance Site (IM-HDSS) [30] in Eastern Uganda. The IM-HDSS area comprises of 65 villages and 15,964 households. Sixty percent of the demographic surveillance area is rural and 38% peri-urban, and most of the population are subsistence farmers [30]. Seventy two percent (72%) of children with CP reside in rural areas [31]. More than half (61%) of the households of children with CP live in extreme poverty, with incomes below the national poverty level (<0.9 US dollars/person/day) [31].

### Study participants

This study was conducted among malnourished C&Y with CP aged 2–24 years, and their primary caregivers residing in the IMHDSS area. Eligibility of C&Y was malnourishment as per WHO growth standards at <-2SD z-score in any of height for age-z scores (HAZ), weight for age-z scores (WAZ), and body mass index for age z-scores (BMIZ) [32]. Eligible caregiver-C&Y pairs were identified after anthropometric assessments as part of the baseline assessments for the Akwenda Intervention Program which aimed to determine the effectiveness of a multidimensional intervention on child health outcomes, caregiver wellbeing, and community attitudes. The program comprised five intervention components; i) caregiver-led training workshops, ii) therapist-led practical group sessions and workshops, iii) provision of technical assistive devices (TAD), iv) goal setting, and v) communication and advocacy for behavioural and social change [29]. Since not all C&Y in the Akwenda study arms were eligible for this study, additional new eligible C&Y with a confirmed CP diagnosis were recruited from disability related non-government organizations operating within the study area, and assigned to either study arms to make up for the required sample size. Caregivers with children receiving any nutrition intervention, and those severely malnourished children (<-3SD) with oedema were excluded, and referred for further management. Eligible caregivers were contacted for voluntary consent.

### Interventions

Two different caregiver-led interventions were tested in this trial, including the positive deviance-nutrition intervention and the parent facilitator training strategy of the Akwenda intervention.

### Positive deviance (PD)-nutrition strategy

The first step of the PD strategy was to conduct a PD inquiry in which well-nourished (positive deviants (PD)) and malnourished children (negative deviants (ND)) and their caregivers were identified from participants of the Akwenda intervention control arm. This was followed by a qualitative exploration (using in-depth interviews) of the lived caregiving experiences of PD caregivers to identify the caregiving practices (S1 Table) that may have positively impacted the nutritional status of their children.

Following the PD inquiry, a list of feeding practices and good foods of caregivers of well-nourished children with CP was generated and used to design the PD guide following the positive deviance-Hearth approach by Save the Children [33]. The designed PD-guide was validated and pretested among previously selected PD caregivers before execution. PD sessions were facilitated by trained PD caregivers selected as PD volunteers, who followed the designed PD guide during all sessions. Session meals were prepared as per the developed PD good food menu (S2 Table), and meals were alternated every other day for each PD group following the PD sessions time table (S3 Table). PD sessions were supervised by the primary researcher and trained field supervisor.

Group sessions included a half hour peer led nutrition and counselling session involved sharing PD caregiver experiences and good motivations to caregiving a child with CP, and a two-hour cooking and child feeding session. PD sessions were conducted within four home steads of participating caregivers chosen as convenient to other caregivers. During the PD group sessions, caregivers contributed locally available foods, and together they prepared meals as following the PD good food menu. Caregivers thereafter fed their children while being monitored by the PD volunteers and session supervisors. At the end of each PD group session, caregivers were given measured portions of locally prepared mixed flour (two table spoons per porridge serving per day) for porridge preparation at home, so as to supplement their home meals during non-session days until the next week group session. Caregivers were also requested not to share the PD foods given with children in neighbouring households. Caregivers who attended group sessions irregularly were followed up by PD volunteers through home visits on subsequent days (S3 Table), and the PD package and food supplements were administered.

### Parent facilitator training strategy

The Parent facilitator training (PFT) intervention was a core component of the Akwenda Intervention Program among children with CP at the IM-HDSS. Parent facilitator training workshops involved training of five primary caregivers of children with CP from the study area as parent facilitators by expert physical therapist for four weeks. Trained parent facilitators trained caregivers of children with CP while following a detailed Lusoga translated manual [34]. Parent facilitator trainings involved 7 sessions that exposed caregivers to understanding CP and how to improve the child’s body functioning, including a session on eating and drinking a healthy diet or nutrition. Sessions on eating a healthy diet were facilitated by trained therapists during the therapist led practical group sessions, who taught caregivers on: i) the seven major classes of nutrients ii) how to prepare a meal from locally available nutrient dense food, and iii) Responsive feeding practices (e.g., frequency of feeding; creating a safe and clean environment for feeding). Therapist sessions were aligned to the same theme as the PFT workshops. All additional eligible children recruited from other CBOs and enrolled onto the PFT intervention arm received only the PFT component and therapist led sessions on eating a healthy diet before randomisation to the two PFT treatment conditions (PFT only and PFT + PD).

### Control condition

The control condition included usual care whereby children with CP in the IMHDSS area access primary healthcare services through public local health centres, although specialised services are limited. Private non-governmental community-based organisations operating in the area provide occasional physiotherapy inform of exercises or occupational therapy, and sometimes wheel chair donations or crutches. There are no specialised nutrition services for children with CP at the IMHDSS, but severe malnutrition cases are often referred to the Iganga Hospital Nutrition Clinic for specialised management.

### Training of staff for intervention

Before initiating the PD intervention, four caregivers selected as volunteer facilitators achieved repeated PD training sessions to deliver the intervention correctly. Two of the caregivers were parent facilitators from the Akwenda intervention study, while the other two were caregivers of well-nourished (positive deviant, PD) C&Y with CP. The training included uniform preparation of PD foods as per the generated PD-good foods menu, and delivery of simplified nutrition education messages and counselling to lay caregivers following a designed and locally translated PD training manual. The PD intervention was run for 3-months (August to October 2022) with four separate PD-group sessions per PD condition (PD only and PFTPD) conducted each month.

### Delivering of the interventions

Four participant home steads convenient for most caregivers were selected for delivery of the PD and PFT sessions. Two of these homesteads were used for sessions by the combined PFT + PD group while the other two were used by the PD only group. Each of the PD only, and combined PFT + PD groups had two independent PD facilitators restricted to each group, with at least 10 caregiver-child pairs participating in each group session.

### Outcome measures

Data was collected at baseline and at follow-up after 3-month. The primary outcome was weight gained at follow-up; secondary outcomes were a change in weight for age z scores (WAZ), and change in BMI for age z scores (BMIZ).

### Data collection procedures

The study team (research assistants) included two clinicians, two therapist who assessed motor severity, and two community mobilisers chosen from the catchment districts. All research assistants were trained on administering the data collection tools, and were proficient in Lusoga the local language. All data collection tools were pretested on four non-participating caregiver-child pairs from the same study setting before examining study participants. Baseline data included information on the caregiver and child’s demographics, gross motor functional classification (GMFCS) level, nutritional status, and caregiver reported feeding related factors. Baseline data enabled comparisons of participants allocated to the four study conditions exploring differences potentially affecting the study outcome.

### Anthropometric measurements

Weight was measured in kilograms(kg) using a calibrated SECA 813 digital scale (Seca Vogel & Halke GmbH &Co., Hamburg, Germany), and readings were recorded to the nearest 0.1 kg. When the child could not stand independently, the body weight was obtained by calculating the difference between the caregiver’s weight while carrying the child and without the child. Height was measured in centimetres (cm) using a portable stadiometer length measuring board with a headboard and sliding foot piece (Shorr Productions, LLC, Maryland, USA) and recorded to the nearest 0.1 cm for C&Y who were able to stand flat-footed and erect. For C&Y with significant musculoskeletal deformities (such as kyphosis, scoliosis or notable deformities in lower-limb flexion) or severe spasticity, height estimates were predicted from the knee height using published validated equations [35]. Body mass index (BMI) was calculated as weight (kg) divided by height in meters squared (m^2^).

### Nutritional status

Weight and height were entered into WHO Anthro (2010) and Anthro-plus (2009) calculators, in which Height, Weight and Body Mass Index for age-Z-scores were automatically calculated up to 19 years of age [36,37]. Nutritional status of each child was assessed on the basis of Z-scores [32] as height-for-age Z score (HAZ), weight-for-age Z-score (WAZ) or BMI-for-age Z score (BMIZ), and only BMI for those above 19 years of age. We defined malnutrition at recruitment when one or more of the anthropometric z-scores was < -2SD.

### Severity of functional limitations

Gross motor function level was assessed by trained therapists using the Gross Motor Function Classification System (GMFCS) [38], which classifies mobility on a 5-point ordinal scale, i.e., varying from level I (mild/independent) to level V (severe/ dependent on assistance for all mobility functions).

### Sample size estimation

Existing evidence pertains to effectiveness of the PD strategy on weight gain only among malnourished typically developed children [25,27,39], while PD effects may differ for children with CP who tend to have varying energy requirements dependent on motor impairment severity [40]. We thus opted to use minimum sample size estimates of a 2x2 factorial design documented by Bonnet 2016 to estimate a main effect in a between-subjects 2x2 factorial design at 95% confidence, with a desired confidence interval width (w) of 2.0. We assumed a planning value of 8 [41] for a within group error variance $\left(\sigma^{2}\right)$, with contrast coefficients $\left(c_{j}\right)$ of 0.5, 0.5,-0.5 and-0.5. The sample size per group was thus approximately 31 [41], and an overall sample size of 124 (4n) caregiver-C&Y pairs assuming no attrition.


$$\begin{array}{c} n_{j}=4{\tilde{\sigma }}^{2}(\sum {\mathrm{\backslash nolimits}}_{j=1}^{k}c_{j}^{2})\;({\frac{z_{\frac{\alpha }{2}}}{w})}^{2}\; \end{array}$$

$$\begin{array}{c} =4(8.0)(\mathrm{.25}+\;\mathrm{.25}+\;\mathrm{.25}+\;\mathrm{.25})({\frac{1.96}{2.0})}^{2}\;\approx 31. \end{array}$$

The sample size was inflated by 10% (n = 136) to cater for possible attrition, although it was not possible to identify additional eligible children with CP for this consideration in the study area at the time of this study. However, efforts to reduce attrition were made, and an overall attrition rate of 6% observed suggested a low level of potential bias.

### Randomisation

This study utilised a pre-existing two-arm participant allocation structure of the Akwenda intervention study was utilised to select study participants. Hence complete randomisation of eligible study participants to the four treatment conditions for the factorial trial was impractical. The caregiver-C&Y pairs of the Akwenda Intervention Program study had been randomized based on two geographical clusters: one receiving the intervention (including PFT) and the other acting as waitlist controls [34]. Random allocation of participants to the PD intervention within each of the two Akwenda study village clusters, and of the additionally recruited children (CBO) was by random numbers generated by an independent individual using the random number function in Microsoft Excel at an allocation ratio of 1:1:1:1 to achieve four same sized groups (n = 31); two PFT conditions (PFT only and PFT + PD) and two non-PFT conditions (PD only or control). Random allocations were kept in a secure electronic folder only accessible to the primary researcher, and made known to the research team at the time of seeking participant consent. Only outcome evaluators were blinded to which intervention the participants received.

### Statistical analysis

Data analysis was by intention to treat and performed using Stata Statistical Software, version 14. Group comparability at baseline was done using descriptive statistics and reported as percentage (%) frequencies (n), mean±sd, and median (IQR) as appropriate. Change in weight status was assessed as a change in: Body weight, WAZ scores, and BMIZ scores after three months. Normality was assessed using the skewness and kurtosis test and visual inspection of the histogram. A two-way full factorial linear regression analysis was done to test differences between C&Y who had received the interventions, and those who had not, and whether an interaction effect existed between the PD and PFT interventions. We investigated with linear regression analysis whether PD or PFT had a main effect on weight gain, while adjusting for baseline weight measurements and relevant baseline participant characteristics (possible confounders). All primary analysis was expressed in 95% two-sided confidence intervals, and a p < 0.05 indicated statistical significance. Participants with missing weight and height data (7/124 (6% attrition)), were excluded from the analysis.

In a factorial design interaction effects may be undetected due to larger sample size requirements [42]. We thus performed an ad-hoc non-factorial analyses ignoring the no interaction assumption of the factorial analysis above, to derive a much stronger conclusion on the effect of the combination of the PFT and PD interventions, using the two-stage procedure [43]. The two-stage procedure was fitting for our sample size (four-fold of the sample size required per arm) [44], and we were able to sequentially determine the effect of the combination condition with adjusted p-values at each stage. At the first stage we compared each of the three experimental conditions with the control condition adjusting for baseline characteristics in a linear regression model with statistical significance at two-sided adjusted p-value 0.017 (α/3). At the second stage we determined whether the combined PFT-PD condition should be recommended by comparing the PFT-PD combination with either intervention conditions separately, at two-sided adjusted p-value 0.033 (2α/3), if either one intervention or the combination was better than the control condition at the first stage. While, statistical significance was at p < 0.05(α), if only the effect of the combined condition was significantly different from the control condition at the first stage [43].

## Results

### Participants

**Fig 1:**
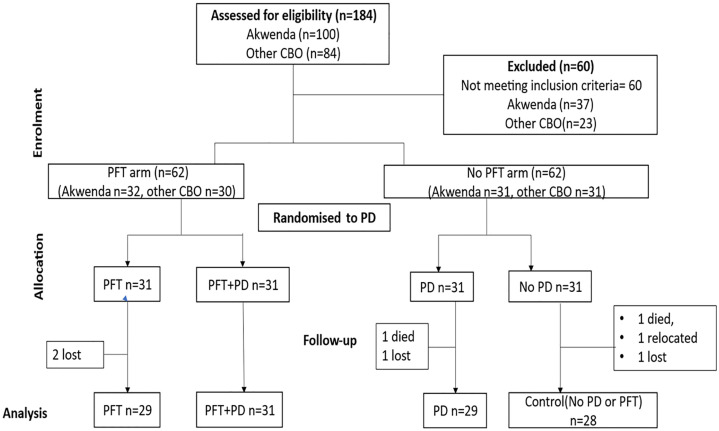
Trial profile. This figure displays the trial profile, including the total number of subjects screened and randomized, group allocation, and an overview of reasons for withdrawal. Abbreviations: PD, Positive Deviance; PFT, Parent Facilitator Trainings; CBOs, Community Based Organisations.

Fig 1 shows the flowchart of all participants. One hundred and twenty-four (124) C&Y with CP were recruited, 63 from the Akwenda study (both the intervention and control arms), and 61 new recruits, allocated evenly between the four groups (n = 31). Overall, 117 participants completed the study, while two children deceased before baseline assessments and 5 children were lost. Statistically significant differences between groups were observed for some characteristics including age and sex of the child, presence of a feeding problem, and number of other children under five years in the household as showed in Table 1. The average participant follow-up duration after baseline assessments was three months and four days (mean = 3.4, SD = 0.3).

**Table 1:** Baseline characteristics of participants by group.

	PFT	PD	PFT&PD	Control	P-value
**Child/Youth characteristics**					
**Nutritional status**					
WAZ (mean±sd)	n = 17 (-3.7 ± 1.2)	n = 15 (-2.1 ± 1.3)	n = 20 (-3.5 ± 1.6)	n = 25 (-3.0 ± 1.8)	0.051
HAZ (means±d)	n = 30 (-3.6 ± 1.7)	n = 29 (-3.2 ± 2.6)	n = 28 (-3.8 ± 1.6)	n = 31(-4.2 ± 1.8)	0.085
BMIZ (mean±sd)	n = 30 (-1.7 ± 1.5)	n = 28(-1.9 ± 1.9)	n = 28(-1.8 ± 2.1)	n = 30(-0.7 ± 1.8)	0.080
BMI (kg/m2)	n = 1(11.1)	n = 2 (13.6, 10.8)	n = 3(17.7,16.6,17.4)		
**Weight (mean**±**sd)**	n = 31(21.1 ± 12.7)	n = 30 (21.6 ± 8.7)	n = 31(21.0 ± 15)	n = 30(15.4 ± 9.5)	0.154
Missing*		n = 01		n = 01	
**Follow-up months** (mean±sd)	3.4 ± 0.3	3.5 ± 0.4	3.3 ± 0.2	3.4 ± 0.3	0.154
**Age** in years (mean±sd)	9.5 ± 5.4	10.5 ± 5.5	9.4 ± 6.7	6 ± 4.0	0.006^a^
**Sex n (%)**					
Male	22(71)	11(36)	16(52)	16(52)	0.049^a^
Female	9(29)	20(64)	15(48)	15(48)	
**GMFCS**^b^ **n (%)**					0.683
Leve1 1	3(10)	1(3)	3(10)	3(10)	
Leve12	4(13)	7(22)	4(13)	4(12)	
Level 3	5(16)	3(10)	3(10)	3(10)	
Level 4	11(35)	13(42)	10(32)	16(52)	
Level 5	8(26)	7(23)	11(35)	5(16)	
**Feeding problem**					0.030^a^
Yes	9(29)	19(61)	13(42)	9(29)	
No	22(71)	12(39)	18(59)	22(79)	
**Self-feeding**					0.455
Yes	11(36)	10(32)	10(32)	9(29)	
No	15(48)	12(39)	18(58)	18(58)	
Unskilfully^c^	5(16)	9(29)	3(10)	4(13)	
**Caregiver characteristics**					
**Age years (mean**±**sd)**	42.3 ± 11.5	43 ± 13.6	40.1 ± 10.2	37.4 ± 9.6	0.209
**Median (IQR)**	40 (32 50 )	43 (30 50 )	42 (29 48 )	38 (30 42 )	
**Relation to child**					0.711
Mother	19(61)	23(74)	23(74)	21(68)	
Grandmother	7(23)	4(13)	5(16)	7(22)	
Father	3(10)	1(3)	2(6)	3(10)	
Other relative	6(6)	3(10)	1(3)	0	
**Education level**					0.540
Primary	20(64)	18(58)	15(48)	17(55)	
Secondary	9(29)	11(35)	9(29)	7(23)	
Tertiary	1(3)	0	3(10)	3(10)	
None	1(3)	2(7)	4(13)	4(13)	
**Occupation**					0.263
Farmer	19(61)	18(58)	19(61)	17(55)	
Housewife	3(10)	1(3)	5(16)	2(6)	
Business	6(19)	4(13)	4(13)	7(23)	
Other None	3(10) **0**	5(16) 3(10)	3(10) 0	16(16) 0	
**Other children mean**±**sd**	1 ± 1.4	2 ± 1.5	1 ± 1/7	1 ± 0.7	0.010^a^
**Household income shs** ^d^					0.261
<100,000	14(45)	18(58)	17(55)	22(71)	
100-200,000	14(45)	12(39)	10(32)	7(23)	
200-500,000	3(10)	0	4(13)	2(6)	
>500,000	0	1(3)	0	0	

Table 1. Baseline characteristics of children/youth and their caregivers. ^a^Pearson chi-square test for comparison of the difference in categorical factors between groups, and ANOVA F-tests for comparison of continuous factors at statistical significance p < 0.05. ^b^Gross-motor functional classification level (1 –5 ). ^c^self-feeding with difficulty like a baby. ^d^Monthly household income in Ugandan shillings, 1 USD ≈ 3,500 Ugandan shillings (shs). *Two children died before baseline weight measurements and one did not turn up.

### Descriptive change in mean weight per group

Table 2 shows the mean change in weight of each of the four groups. A significant increase in mean weight was observed for the whole C&Y group mostly driven by those in the PD only and the combined PFT + PD groups with a significant increase in weight at follow up. The increase in weight among C&Y in the PFT only group was not statistically significant.

**Table 2:** Descriptive change in mean weight per group.

	N	Baseline Weight (W_1_) Mean(SD)	Weight at follow-up (W_2_) Mean(SD)	W_2_-W_1_ Mean(SD)	p
**All children**	117	20.1(12)	20.5(12)	0.36(1.0)	<0.01*
**PFT**	29	21.9(13.0)	22.1(13.0)	0.15(1.2)	0.517
**PD**	28	22.03(8.6)	22.7(8.7)	0.65(0.9)	0.001*
**PFT + PD**	31	21.03(15.2)	21.6(15.7)	0.53(1.1)	0.008*
**Control**	29	15.5(9.6)	15.6(9.8)	0.10(0.7)	0.536

Table 2 displays the mean weight at baseline (W_1_), weight after three months (W_2_), and the change in mean weight within each of the four groups before adjustment for baseline characteristics. Paired t-tests were used to test the difference between the first and last weight measurements within groups. *Statistical significance at p < 0.05.

### Interaction Effect

**Fig 2:**
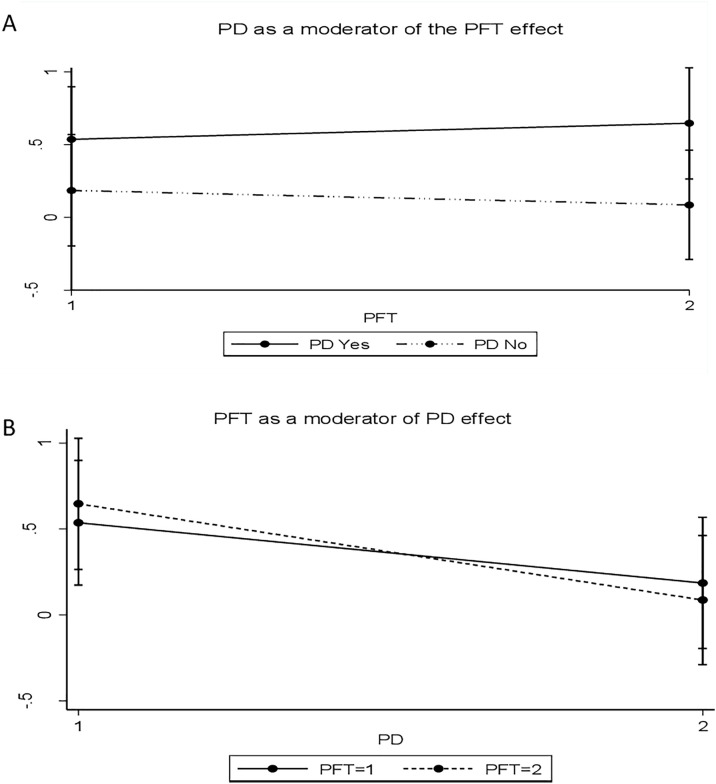
The figure shows a graphical presentation of the interaction between PD and PFT interventions. A. shows PD moderating the effect of PFT. B. shows PFT moderating the effect of PD.

The interaction effect between the PD and PFT interventions on weight gain was not statistically significant (interaction coefficient = -0.18, 95% CI -0.92,0.57, p = 0.641), although evident with a graphical presentation (Fig 2).

### Effect of the PD intervention on nutritional status

After adjustment for baseline characteristics in a multiple linear regression model (Table 3), C&Y who received the PD intervention significantly gained more weight by 520g than those who did not receive PD (adjusted coefficient = 0.52, 95%CI 0.16,0.88, p = 0.005), and their BMIZ scores significantly improved by 0.65 scores more than those who did not receive PD (adjusted coefficient = 0.65, 95%CI 0.35,0.94, p=<0.01). The usual response to PD interventions is a weight gain of>900g over a three months period. In our study, weight gain > 900g after three months was observed among 42% (25/59) of the C&Y who received PD, and among 29% (17/58) of those who did not receive PD. In addition, there was no statistically significant difference in the change in WAZ scores between those who received PD and those who did not receive PD (adjusted coefficient = 0.10,95%CI -0.24,0.37, p = 0.663).

**Table 3:** Main effects of PD and PFT on nutritional status after adjusting for participant baseline characteristics.

	Freq. n (%)	Unadjusted Coefficients(95%CI)	Unadjusted p-values	Adjusted coefficients(95%CI)^1^	Adjusted p-values
**Weight gain**					
**PD**					0.005*
Yes	59(50)	0.47(0.10,0.83)	0.014	0.52(0.16,0.88)	
No	58(50)	Ref			
**PFT**					0.240
Yes	60(51)	-0.01(-0.40,0.37)	0.953	0.21(-0.14,0.56)	
No	57(49)	Ref			
**WAZ score**					
**PD**					0.663
Yes	33(46)	0.16(-0.17,0.48)	0.341	0.10(-0.24,0.37)	
No	38(54)	Ref		Ref	
**PFT**					0.006*
Yes	35(49)	0.20(-0.12,0.52)	0.214	0.42(0.14,0.56)	
No	36(51)	Ref		Ref	
**BMIZ score**					
**PD**					<0.01*
Yes	54(49)	-0.57(-0.91, -0.24)	0.001	0.65(0.35,0.94)	
No	57(51)	Ref		Ref	
**PFT**					0.067
Yes	56(50)	-0.12(-0.47,0.23)	0.494	0.27(-0.02,0.56)	
No	55(50)	Ref		Ref	

Table 3. Multiple linear regression modelling of weight gain, weight for age z-scores and body mass index for age z-scores at follow-up. ^1^Adjustment for baseline child and caregiver characteristics: age, sex, weight at baseline, GMFCS level, feeding problem, self-feeding, caregiver education. *Statistical significance at p < 0.05.

### Effect of the PFT intervention on nutritional status

There was no statistically significant difference in weight gain between C&Y who received PFT and those who did not (adjusted coefficient = 0.21,95%CI -0.14,0.56, p = 0.240). Similarly, there was no statistically significant difference in the change in BMIZ scores between those who received the PFT intervention and those who did not receive PFT (adjusted coefficient = 0.27,95%CI -0.02, -0.56, p = 0.067). However, the change in WAZ scores significantly increased by 0.42 scores more among those who received the PFT intervention than those who did not receive PFT (adjusted coefficient = 0.42, 95%CI (0.14,0.56, p = 0.006), while (Table 3).

### Effect of combining the PFT and PD interventions on nutritional status

Table 4 shows results of the ad-hoc analysis using non-factorial manipulations with the two-stage procedure [43,44] to determine the effect of the combination while ignoring the no interaction assumption of factorial analysis. At stage 1, outcomes of each of the three groups were individually compared to the control group, and at stage two each of the PD only and PFT only groups were compared with the combined PFT + PD group.

**Table 4:** Two stage procedure for non-factorial analysis of weight status by comparison of Arms: PD, PFT, PFTPD and Control.

	Weight gain		WAZ change		BMIZ change	
	**Adjusted coefficients**^1^ **(95% CI)**	**Adjusted p-values**	**Adjusted coefficients**	**Adjusted** **p-values**	**Adjusted** **Coefficients** **(95%CI)**	**Adjusted** **p-values**
**Stage 1: Comparison with control condition** ^**2**^						
PFT vs Control	0.39(-0.18,0.97)	0.142	0.4(-0.9,0.9)	0.109	0.1(-0.4,0.5)	0.723
PD vs Control	0.72(0.10,1.34)	0.012*	0.0(-0.5,0.5)	0.998	0.4(-0.6,0.9)	0.087
PFTPD vs Control	0.77(0.22,1.32)	0.006*	0.4(-0.01,0.8)	0.055	0.9(0.4,1.3)	<0.01*
**Stage 2: Comparison with combination condition** ^**2**^						
PFT vs PFTPD	-0.38(-0.85,0.09)	0.116			-0.8(-1.2,-0.4)	<0.01**
PD vs PFTPD	-0.05(-0.54,0.44)	0.847			-0.5(-1.3,-0.4)	0.051

Table 4 shows the two-stage procedure for a non-factorial analysis of a two-by-two factorial design. ^1^Coefficents after adjustment for baseline characteristics at two-sided statistical significance level p < 0.05(α). ^2^Three group comparisons in stage 1 at adjusted alpha p < 0.017(α/3). ^3^Stage two comparisons at adjusted alpha p < 0.033 (2α/3) since the combined condition but not the PFT condition was better than the control condition. ^3^Stage two comparisons by BMIZ at p < 0.05 since the combined condition was better than the control at stage 1. Statistical significance *p < 0.017, **p < 0.05

Compared to the control group, C&Y in the combined PFT + PD group significantly increased their weight by 770g (coefficient = 0.77 95%CI 0.22, 1.32, p = 0.006), and also significantly increased the change in BMIZ scores by 0.9 scores (adjusted coefficient = 0.88 95%CI 0.43, 1.34, p < 0.01). No statistically significant differences were observed in the change in WAZ scores between C&Y who received the combined interventions and the control treatment. When the combined PFT + PD group was compared with the PFT only and PD only group, we found that those in the PFT only and PD only groups insignificantly decreased their weight by 380g and 5g respectively. Furthermore, a significant decrease in BMIZ scores of 0.8 scores more was also found among those in the PFT only group compared to those in the combined PFT + PD group (adjusted coefficient = -0.8 95%CI -1.2, -0.4, P=<0.01).

## Discussion

In this study, the PD intervention significantly improved the nutritional status of C&Y with CP by weight gain, and increased BMIZ scores. Meanwhile the PFT intervention had insignificant improvements in weight gain and BMIZ scores, although improvements in WAZ scores were statistically significant. Combining both interventions significantly increased weight gain and BMIZ scores more than not providing any intervention, or providing the PFT intervention alone.

Our findings are similar to those reported by previous studies among malnourished typically developed children in Zambia [27] and Burundi [25] where 100% and 87% adequate weight gain was respectively observed among children participating in a PD intervention. The PD nutrition strategy involves dietary modification while using locally available nutrient dense foods to improve malnutrition [28]. Nutrition interventions that involve dietary modifications have previously been indicated to have better effects on the nutritional outcomes of children with CP [17]. Our PD strategy had a dietary modification component in which locally prepared foods with varieties that define a healthy diet were utilised [45]. This may have contributed to weight gain of our C&Y participants. Kajuju et al in Kenya similarly reports significant changes in weight status with improved protein and vitamin A intake after providing a locally fortified millet porridge to children with CP [21]. However, we cannot reliably ascertain nutritional value of our locally prepared PD foods since we did not assess dietary content and body composition changes of participants in this study. We thus highly recommend further investigations in this regard.

Adequate weight gain in PD interventions among typically developed children has been reported as weight gain of ≥ 900g within 3 months [26], and a study in Zambia reported 100% response [27]. In our study, the effect size of 520g from PD among C&Y with CP did not meet the typical cutoff, although it still indicates a clinically significant benefit compared to those without PD. While 42% (25/59) of those on PD attained adequate weight gain ≥ 900g after three months, only 29% (17/58) of those without PD attained this cutoff. Furthermore, a PD comparative study in Uganda reports 18.7% reduction in underweight after a PD intervention [39], yet there was no significant change in underweight among our PD participants. These findings highlight the need to adapt the PD intervention for children with special needs like cerebral palsy. The lower response rate and weight gain in the CP population may be due to the unique nutritional challenges associated with cerebral palsy [46]. Differences in required caloric intake per ambulatory status among individuals with CP may cause slower weight gain with similar nutritional management [40,47]. Regular nutritional monitoring is thus crucial among individuals with CP, and the need to adjust the PD intervention, and other nutritional strategies to ensure effectiveness among those with special needs like CP.

Additionally, most of our participants were much older (2–24years) than typically developed children (under 5 years) who ordinarily participated in previously reported PD interventions [25,27]. Malnutrition among typically developed children is usually managed at age five years and below, and improved growth and survival outcomes have widely been reported [48,49]. While among children with CP, malnutrition persists past childhood to young adulthood with excessive related mortality below age 10 years [12,13]. This indicates a need to include transition plans for older children (above five years) with cerebral palsy in existing nutrition guidelines in order to improve survival prospects through ongoing support and care.

In this study, the difference in weight gain between C&Y who received the parent facilitator training (PFT) intervention, and those who did not receive PFT was not statistically significant, although the weight for age z-scores of those who received PFT highly significantly improved more than those who did not receive PFT. Our findings contradict those from a low-cost caregiver training intervention in Bangladesh to improve caregiver feeding practices were inconsistent improvements on growth of participating children with CP were observed [50]. Similarly in Ghana, a caregiver training program to improve feeding practices showed no improvements in anthropometric status of children with CP [51]. These findings suggest that physical therapy may not immediately, apparently influence nutritional outcomes, although the improvement in weight for age scores may indicate indirect effects on nutritional outcomes such as changes in energy expenditure, muscle mass and overall health.

Although our PFT trainings focused on improving child functional outcomes, and caregiver wellbeing, sessions on physical support during feeding and provision of a healthy diet were also included. This may also partly explain the observed increase in WAZ scores among children who received PFT in this study. Additionally, half of the participants in the PFT arm had received the full Akwenda program package before recruitment to this study. The Akwenda intervention showed great improvements in child gross motor outcomes and selfcare skills [34], with caregivers displaying a positive change of attitude towards their children all of which may have led to improved feeding practices and nutritional status. Additionally, relatively prolonged exposure to physio-therapy as part of the Akwenda intervention may have led to reduced body fat content, but increased muscle mass of participants, thus improving weight for age without necessarily increasing weight gain or BMI scores in this group [52]. These sub-group differences may explain the observed significant effect of PFT on WAZ and not on other weight indices among PFT participants. Nonetheless, body composition of C&Y was not assessed in this study to justify this judgement.

Furthermore, insignificant effects of our PFT intervention on overall weight gain and BMIZ scores among older children, may signal a reluctance of caregivers on improving their children’s functional abilities, including assisted feeding as they grow older, despite the acquired knowledge and skills. Caregiver health seeking for child functional abilities, and support with caregiving roles have been reported to diminish as children with CP transition into adolescence with concerns about remaining actively involved in their children’s care with higher caregiver burnout levels [53–55].

In this study, C&Y who received both the PFT and PD interventions significantly gained more weight and increased their BMIZ scores more than those who did not receive any of the PD or PFT interventions, and those who received only the PFT intervention. Improving disability has been indicated to ultimately reduce the risk of malnutrition and vice-versa [10]. However, interventions reported to focus on improving the level of impairment or feeding abilities of children with CP without a dietary component, have previously not shown significant effects on child nutritional outcomes [50,51]. On the other hand, a recent intervention in China that focused on improving dietary intake of children with CP indicated improved gross motor function [20]. Intense physical rehabilitation increases energy requirements of children with CP [56], and thus adequate dietary intake should be incorporated into physical rehabilitation strategies for better overall health outcomes of C&Y with CP. These study findings highlight the complexity of growth and weight metrics among children with CP and the importance of considering multiple factors when designing nutrition interventions. Motor severity is a key predictor of nutritional status and can guide nutritional planning among children with CP. However, in our study, we did not prespecify analysis of intervention response by the level of motor severity to report on this. Future studies may be designed in consideration of these aspects so as to report on whether the effects of the interventions differ across the different levels of motor severity so as to guide targeted nutrition strategies in this population.

A multidimensional approach that incorporates both nutrition and functional improvements might be the most effective strategy to malnutrition among children with CP.

### Study strengths and limitations

To the best of our knowledge, this is the first experimental study to test the effect of the positive deviance strategy on the nutritional status of children and youth with CP in low-income countries (LICs), and the observed significant effect of the positive deviance (PD) intervention on the nutritional status of C&Y with CP can be interpreted to mean that the PD approach can be effectively adapted to any setting with malnourished children with CP, as has successfully been internationally applied among typically developed children. The factorial trial design allowed the ability to answer multiple research questions about two interventions in one trial using a relatively smaller sample.

However, this study had some limitations. Participant selection was primarily within the existing Akwenda study two arm structure, and thus allocation of participants to study conditions was not completely random, which increased the potential for non-equivalent study groups and a high risk of bias. However, the difference in groups observed in some baseline factors was adjusted for during statistical analysis to determine the independent main effects of the PD and PFT interventions. Furthermore, we did not establish the dietary content of our PD foods to estimate the required protein and energy requirements for catchup growth of participating C&Y before commencing the interventions [56]. Ascertainment of dietary content of some of our PD foods from available food composition soft-wares like the modified Nutrisurvey 2007 (EBISpro, Germany), was not possible as some local foods used are not included, and available food composition tables do not contain entries for composite dishes used in our PD menu [57]. Standardisation of the preparation and serving rations of our locally combined PD foods thus warrants future studies before future adaptations.

## Conclusion

We conclude that the PD intervention significantly improves weight gain of C&Y with CP while the PFT intervention significantly improves weight for age z scores among younger children. We found that combining the PFT and PD interventions provides better effects on nutritional status than not providing any intervention, or providing the PFT intervention alone. Based on these findings we recommend the integration of locally derived nutrition specific interventions into existing community based therapy and nutrition rehabilitation programs, to improve the nutritional outcomes of C&Y with CP in a low resource setting.

## Supporting information

S1 Table Feeding practices of positive deviant caregivers after a qualitative inquiry. ^a^Common sauce used by 10 PD caregivers include, groundnuts (3), dodo (amaranthus) (4), silverfish (2), beans (4), eggplants (aubergine) (3), kulekula (oysternuts) (2), ovacado (4).
(DOCX)

S2 Table Positive Deviance Good Food Menu showing a combination of foods used per PD session meal. Healthy porridge A and B and Main meal food combinations were alternated between weekly session days.
(DOCX)

S3 Table Group Sessions time table for monthly PD session activities. ******Non-PD session days during which follow-up home visit were conducted.
(DOCX)

S1 Checklist Consort Checklist. (DOCX)

S1 Protocol Trial Protocol. (DOCX)
