# Supplementary material for: Effectiveness of the Positive deviance and parent facilitator training strategies on the nutritional status of children and youth with cerebral palsy: A quasi-randomised trial with a factorial design
Source: PLOS Glob Public Health. 2025 Aug 19;5(8):e0005027. doi: 10.1371/journal.pgph.0005027 (PMC12364356; doi:10.1371/journal.pgph.0005027)
Supplement: S1 Table — aCommon sauce used by 10 PD caregivers include, groundnuts (3), dodo (amaranthus) (4), silverfish (2), beans (4), eggplants (aubergine) (3), kulekula (oysternuts) (2), ovacado (4). (DOCX) [file pgph.0005027.s001.docx]

**S1 Table: Themes derived from 12 Positive Deviant caregivers**

| **Theme** | **Sub-theme** | **Category (n)** |
| --- | --- | --- |
|  |  |  |
| **Positive Deviants (PD)** |  |  |
|  |  |  |
| Feeding practices | Responsive | Feeding relationship (10), attending to feeding needs (10), Family meal integration (10), Hygienic feeding (4) |
|  | Adaptive | Focus on child feeding abilities (5), special feeding routine (5), special feeding utensils (4), special food preparation (2) mealtime creativity (6) |
|  |  |  |
|  | Food type | Fruits (5), Tubers and plantains (9), grains (11) sauces (10) ^a^, porridge (5), dry tea (3), milk (3), snacks (1) |
| Social support | Family support | Spouse support (3), siblings support (3), other relatives (04) |
|  | Health care support | Medical care (10), traditional and medical (2), physical rehabilitation (6), home based physical rehabilitation (6), feeding knowledge (4) |
| Coping mechanisms |  | Emotional coping (7), adjust parenting roles (11), self-satisfaction (12), focus on child selfcare abilities (6) |

Table S1: Themes derived from positive deviant caregivers. ^a^ Common sauce used by 10 PD caregivers include, groundnuts (3), dodo (amaranthus) (4), silverfish (2), beans (4), eggplants (3), kulekula (oysternuts) (2), ovacado (4).
