# Supplementary material for: Effectiveness of the Positive deviance and parent facilitator training strategies on the nutritional status of children and youth with cerebral palsy: A quasi-randomised trial with a factorial design
Source: PLOS Glob Public Health. 2025 Aug 19;5(8):e0005027. doi: 10.1371/journal.pgph.0005027 (PMC12364356; doi:10.1371/journal.pgph.0005027)
Supplement: S2 Table — Healthy porridge A and B and Main meal food combinations were alternated between weekly session days. (DOCX) [file pgph.0005027.s002.docx]

**S2 Table: Positive Deviance Good Food Menu**

| **PD good food** | **Food content** | **Frequency per session and amount** |
| --- | --- | --- |
| 1. Healthy snacks | Ripe yellow bananas or mangoes | At least one before main meal |
| 1. Healthy porridge A | Roasted millet, soya, maize, red g.nuts/oysternuts, ground silver fish and egg shells, small white beans and rice, sugar and water | At least Twice a day. A half-litre cup in the morning and evening hours**.**  T table spoons of mixed flour for each porridge serving. |
| 1. Healthy porridge B | Plain millet flour or mixed porridge A flour, kisubi ripe bananas, spear grass, water, no sugar. | At least Twice a day. A half-litre cup in the morning and evening hours**.**  Two table spoons of flour for each porridge serving. |
| 1. Main meal food combinations | 1.Posho+g.nuts+silver fish+amaranthus  2.Sweet potatoes+ beans + silverfish  3.Matooke+g.nuts+amaranthus(dodo)  4. Posho +matooke + oysternuts  5. All meals accompanied with water | One main course meal prepared during each day session. |
