# Supplementary material for: Effectiveness of the Positive deviance and parent facilitator training strategies on the nutritional status of children and youth with cerebral palsy: A quasi-randomised trial with a factorial design
Source: PLOS Glob Public Health. 2025 Aug 19;5(8):e0005027. doi: 10.1371/journal.pgph.0005027 (PMC12364356; doi:10.1371/journal.pgph.0005027)
Supplement: S3 Table — ******Non-PD session days during which follow-up home visit were conducted. (DOCX) [file pgph.0005027.s003.docx]

**S3 Table: PD Monthly Group Sessions time table**

******PD Non-session days PD session days
