# Supplementary material for: Effectiveness of the Positive deviance and parent facilitator training strategies on the nutritional status of children and youth with cerebral palsy: A quasi-randomised trial with a factorial design
Source: PLOS Glob Public Health. 2025 Aug 19;5(8):e0005027. doi: 10.1371/journal.pgph.0005027 (PMC12364356; doi:10.1371/journal.pgph.0005027)
Supplement: S1 Protocol — (DOCX) [file pgph.0005027.s005.docx]

**Effectiveness of the positive deviance and parent facilitator training strategies to malnutrition among children and youth with cerebral palsy in Eastern Uganda: Trial Protocol**

**Introduction:**

In an earlier study done in Uganda by Kakooza Mwesige et al, a high rate of premature mortality among children with CP at the Iganga Mayuge health and demographic surveillance sites, was suspected following the noticeable decrease of CP prevalence with age. High rates of malnutrition, frequent comorbidity and severe impairment were alleged to be contributory. The high prevalence of malnutrition in the CP child population in Uganda is further reiterated by Kakooza Mwesige et al among a clinical CP child cohort at the Mulago regional referral hospital, as 52% especially among those who are five years or older [^1^](#_ENREF_1) . Malnutrition is a multifaceted condition which if not managed effectively may affect the overall health outcome of a child with CP, including their comorbidity status and impairments progression. However, efforts to curb malnutrition although evident among the typical child population, are less proven among children with disabilities including CP [^2^](#_ENREF_2) [^3^](#_ENREF_3)^,^ [^4^](#_ENREF_4) [^5^](#_ENREF_5). Children with CP spend most of their time with caregivers, hence management of CP child malnutrition should actively involve primary child caregivers.

The Positive deviance (PD) approach is one of the caregiver led strategies that has revealed nutritional success in the typical child population in some African settings [^6^](#_ENREF_6)^,^ [^7^](#_ENREF_7). The PD approach operates on the premise that in most population distributions, for example in the field of nutrition, there is a statistically defined portion of the highly malnourished population who deviate positively from the population mean (outliers), and fall within the range of the normally nourished (positive deviants)[^8^](#_ENREF_8). This study thus seeks to evaluate the effectiveness of positive deviance and parent facilitator training strategies to malnutrition in the CP child and youth population, so as to enhance health promotion and survival in this vulnerable population.

This study will be hinged on the cerebral palsy in Uganda risk factors, intervention and epidemiology (CURIE) research project which has implemented at the Iganga Mayuge health and demographic surveillance sites (IM-HDSS) in eastern Uganda since 2015. The CURIE project conducted the CP prevalence study in 2015 identifying 97 children with CP through a three stage screening process[^9^](#_ENREF_9). Ninety seven age and sex matched controls without CP were also recruited in 2015 for the CURIE follow-up study utilizing the CURIE prevalence study findings as baseline, and later conducting follow-up assessments in 2019. Using similar tools, gross and fine motor function, and nutrition and clinical examination assessments were made at the two points of assessment in 2015 and 2019 among the same study population of children with CP and their matched controls. An intervention study (Akwenda) was there after conducted by the CURIE study team between 2021 and 2022 in which effectiveness of a comprehensive intervention among surviving children with CP was tested. The Akwenda intervention included a Carer-to-Carer approach (parent facilitator trainings) on child functional outcomes, and caregiver quality of life, and a communication and advocacy intervention to improve community perceptions towards CP. The current study will utilize the CURIE intervention study arms to answer its intended objectives while using the same study population.

**Methods:**

**Study setting:** The study will be conducted within the districts of Iganga, Mayuge and Bugweri in eastern Uganda.

**Study design:**

This will be a 2x2 factorial quasi-randomized controlled trial assessing the effects of the Positive deviance (PD) (Yes/No) and Parent facilitator training (PFT) (Yes/No) strategies within four experimental conditions: i) Both PD and PFT, ii) Only PD, iii) Only PFT, iv) Neither PD and nor PFT (controls).

**Study population:**

The study population will include caregivers of malnourished children and youth aged 2-24 years old with a confirmed diagnosis of CP originally participating in the Akwenda intervention study. Additional new children with a confirmed CP diagnosis will be recruited from the study area to meet the sample size requirements.

Exclusion criteria: Caregivers registered in other nutrition interventions and those with severely malnourished children that require life-saving nutrition management will be excluded from participating in the study.

**Sample size estimation:**

1. **Sample size estimation in a between subjects 2x2 factorial design (Bonnet,2016)**

**
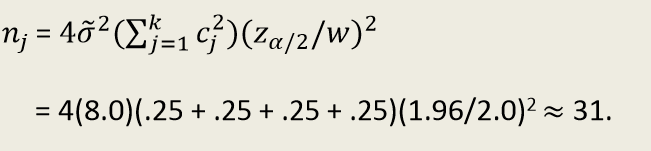
**

In order to estimate a main effect in a between-subjects 2x2 factorial design at 95% confidence, at desired confidence interval width of 2.0, and a planning value of 8.0 for the average within group error variance, the contrast coefficients are 0.5, 0.5,-0.5 and-0.5, and the sample size required per group is approximately 31.

1. **Sample size estimation using two proportions:**

The PD program has been reported to increase weight of ≥200g in 12 days among 87% of participants in Burundi [^10^](#_ENREF_10). Based on the prevalence of malnutrition (<-2sd in any of HAZ,WAZ,BMIZ) among the CURIE CP population based cohort reported as 64% as per the 2015 assessments. Assuming the previously reported effect of the PD program, we hope to reduce malnutrition in the CP child population to about 29%, at α =0.05, and power (1-β) =0.8. After imputing these parameters into a Stata program to estimate sample size for individual randomization, of a two proportions test, the estimated total sample size is 62: Hence 31 participants per each of the four arms. Therefore for a 2x2 factorial trial, assuming a 1:1:1:1 allocation, 124 participants will be required for the study with 32 participants in each of the four conditions.

Participants in this study will include eligible caregivers of children with CP participating in the wider CURIE intervention study (Akwenda). In order to account for those that may not be malnourished from the CURIE study sample, additional caregivers of malnourished children with CP will be recruited from community-based organizations (CBOs) operating within the study setting including EKISA ministries and MUSANA community health centre. The CURIE intervention study has got two arms (PFT and Control) separated as village clusters based on geographical proximity and balanced by age, and GMFCS level. This study will utilize the available CURIE intervention clusters to avoid contamination. Details of participant recruitment from different CBOs are displayed below.

**Participant recruitment:**

**a)Mapping of organisations dealing with children with CP within the Iganga, Mayuge and Bugweri districts as catchment areas.**

Implementing health and research organizations with specialty in community based research and rehabilitation of childhood disabilities including CP, and operating within the study catchment area will be identified and approached for participant identification with the help of community leaders and other key informants. Currently, three organizations dealing with children with CP are known to operate in the study area, and these include: i) the CURIE research project that identified and followed up 97 children with CP within the IMHDSS (described in sub-study 1) in 2015[^9^](#_ENREF_9) to 2019, ii) the Musana Community Health Center (MCHC) a 24-hour facility operating in Iganga district since February 2016 ([www.musana.org](http://www.musana.org)), and EKISA ministries. The MCHC offers affordable services in pediatrics, occupational and physical therapy, minor surgeries, maternity, and community outreach programs within the most rural areas of Iganga. The MCHC also receives patients from neighboring districts including Bugiri, Mayuge and Bugweri.

1. **Identifying children with CP and their caregivers:**

Lists of all children with a confirmed diagnosis of CP will be compiled by a trained community mobiliser from databases of selected community based rehabilitation and research entities (CURIE) as well as additional case finding through community leaders, to make the required number from which to screen the study participants. These lists will include full names of the child and caregiver including contact details and home addresses. Children with missing caregiver contacts or home addresses will be omitted. Caregivers will be traced through phone contacts and household visits to confirm physical address, presence of the CP child and to eliminate participant duplication. Through home visits, voluntary consent for study participation will be sought from all caregivers of children with CP. Unique identification numbers will be assigned to consented caregivers and their children to be enrolled in the study.

1. **Screening for CP child malnutrition:**

To screen for malnutrition, CP child anthropometric measurements will be recorded by a trained clinical officer using standardized WHO protocols[^11^](#_ENREF_11). Standing height will be measured for children > 5 years and recumbent length will be taken for children <5 years, both measured to nearest 0.1cm. Knee height will be recorded for all children to the nearest 0.1cm using a flexible tape measure and used to determine an estimated body height for those who cannot stand, by using the Stevenson formula[^12^](#_ENREF_12). Weight will be measured and recorded to the nearest 0.1kg using a SECA 813 digital scale (seca Vogel & Halke GmbH &Co., Hamburg, Germany). Children who can’t stand independently will first be weighed together with their caregiver and then the weight of the caregiver alone subtracted to obtain the child’s weight. Following WHO best-practice procedures, mid Upper Arm Circumference (MUAC) for all children will be measured using a standard UNICEF MUAC tape.

**Defining malnutrition**:

Anthropometric indicators will be constructed according to WHO growth standards based on weight, height/length, age and sex[^11^](#_ENREF_11) and converted into Z-scores using the WHO Anthro[^13^](#_ENREF_13) and Anthro-Plus to calculate the nutritional status for children within the 2-19 years age range. For those older than 19 years, the LMS method will be used to determine their weight, height and body mass index (BMI) by age and sex standard deviation scores[^14^](#_ENREF_14). Children with a Z-score of < -2 SD in any of the nutritional indicators will be defined as having Malnutrition. Classifications for MUAC will be made by comparing the arithmetic mean of the two measurements (i.e. the “gold standard” measurement) against case defining thresholds for moderate acute malnutrition (MAM) i.e. for children, MUAC < 12.5 cm and severe acute malnutrition (SAM) MUAC < 11.5cm[^15^](#_ENREF_15)^,^ [^16^](#_ENREF_16). For adult (>18years) thresholds, MAM MUAC <23.0 cm and SAM MUAC <21.0cm[^17^](#_ENREF_17). The caregiver of a child or adolescent who is identified as MAM will be enrolled for the intervention study consecutively to make-up for the required study sample (124 caregivers). Caregivers of children or adolescents identified as serious SAM will be counselled about the life saving importance of early management of malnutrition and thereafter referred to the nearest treatment site based on ministry of health guidelines [^18^](#_ENREF_18). Anthropometric findings for those enrolled will be recorded as baseline for the intervention study.

**Outcome measures**

**Primary outcome**: Nutritional status: The primary outcome measure will be a weight gain of >200grams in 12 days[^10^](#_ENREF_10) and ≥ 900g within 3 months.

**Secondary outcomes** will include change in anthropometric Z scores for any of weight, height and BMI, and MUAC classification measure, and Caregiving. The caregiver outcome measure will include a score of the questionnaire evaluating caregiver’s basic nutrition and feeding knowledge before and after the intervention.

**Interventions:**

1. **Positive deviance (PD)-approach:**

The PD-program will be tested among caregivers of identified malnourished children with CP. The positive deviance inquiry (PDI) report detailing successful feeding and caregiving practices from positive deviant families of children with CP (from previous qualitative assessments) will be used to develop the positive deviance (PD) nutrition intervention program.

**Positive deviance nutrition rehabilitation intervention:**

The PD-program will run for 3-months with 2 weeks sessions (12 days) conducted in each month. Each day sessions will include a half hour education session and a one and half hour peer led cooking session. Education sessions will be introduced through situational discussions and illustrated by an educational medium such as an appropriate game or a message. Caretakers will learn how to rehabilitate their malnourished children under the supervision and the support of positive deviant caregivers. Children will be nutritionally assessed by trained nutrition assistants or clinician on the 1^st^, 6^th^, and 12^th^ day of the sessions and after three months.

Caregivers will learn how to prepare nutrient-rich meals for their children from locally available food identified during the PD-inquiry, and good feeding and hygiene practices. Caregivers will together prepare their own food menus based on the PD-inquiry good foods or what they can conveniently access but of equal nutritional value. The menus will include foods which are locally available and affordable and of balanced nutrient value. Food menus will be repeated at least twice within each monthly sessions. However, because the participating children in the PD program are malnourished, total calorie and protein intake in the menus will exceed the daily normal requirement. For sustainability, participating caregivers will contribute the foods and required ingredients during the sessions from those locally available. Caregivers will also learn how to be able to identify child malnutrition through clinical signs, and other common preventable illnesses in childhood. Names and anthropometric data from the sessions will be recorded so as to track changes in nutritional status[^6^](#_ENREF_6)^,^ [^7^](#_ENREF_7).

1. **Parent facilitator trainings.**

The Parent facilitator training (PFT) intervention is part of the wider CURIE community-based intervention study among children with CP at the IM-HDSS. The CURIE intervention study consists of an intervention and control group (1:1 ratio) selected from a previous cohort of surviving children with CP recruited in the CURIE follow-up study and other eligible children with CP selected from the Iganga Mayuge districts CBOs to make-up the desired sample size of 90 children with 45 children per arm. Ethical approval of the CURIE intervention study has been obtained at reference (SS 5173)

The parent facilitator training programme is based on the Malamulele Onward Carer-2-Carer program (https://www.malamuleleonward.org/online-resources/c2c-materials) in South Africa. The program is designed to be delivered by parents/caregivers of children with CP in seven workshops that aim to improve CP child’s participation and activities of daily living (e.g, eating, dressing, and play).

**Training parent facilitators**: Four primary caregivers of children with CP from Iganga are trained as parent facilitators by physical therapist. The training involves four weeks of training from a master trainer from Malamulele Onward, herself a parent of a child with CP. Each workshop is approximately two hours long and includes between 6-10 caregivers. Each workshop is led by two parent facilitators who work as a team. To ensure quality and consistency of the workshops, the Parent Facilitators follow a detailed manual adapted from the Malamulele Onward Carer-2-Carer Programme translated into *Lusoga*, the local language. For each workshop the Parent Facilitators have a workshop kit, which includes display materials. No therapist will be involved in the monthly parent-led workshops. Two weeks after the workshops, caregivers and their children will attend a therapist-led practical group session which will have the same theme as the parent-led workshop.

**Parent facilitator-led sessions:** Parent facilitator sessions are held at a convenient community center. At the start of parent facilitator led sessions, caregivers are given a pretest on their knowledge about CP and caring for a child with CP including basic nutrition knowledge. A similar post –test assessment will be done at the end of the training program. Parent facilitator trainings involve 7 sessions that expose caregivers to understanding CP and how to improve body functioning: session1: what is CP? session2: CP as a way of life, Session 3: Getting my child’s body ready to move, Session 4: Eating and drinking a healthy diet, Session 5: communication, Session 6: Play, and Session 7: Central visual impairment. The therapists conduct regular monitoring and evaluation site visits to rural communities where the parent facilitators are working for ongoing training and up-skilling[^19^](#_ENREF_19).

**Data collection:**

The data collection team will consist of a clinician to take anthropometric assessments, therapists to assess for CP severity, research assistants equipped with skills in data collection, and community mobilisers chosen from the catchment districts.

Once the caregiver-child pairs are enrolled, the research assistants will use a designed locally translated structured questionnaire to record identifying and demographic data: i) the child’s demographics, feeding difficulties, other reported health conditions and recorded anthropometry ii) the primary caregiver’s socio-demographic and economic characteristics. Severity of cerebral palsy will be assessed by trained therapists using the Gross Motor Function Classification System[^20^](#_ENREF_20). Feeding difficulties will be assessed based on parent /caregiver reports using on responses to questions on feeding extracted from an informal questionnaire previously designed by the CURIE study for clinical assessments. Parental/caregiver reports have historically been used as a measure of feeding and or oral motor status in individuals with CP.

**Randomization and blinding:**

**Randomization of participants from the CURIE (Akwenda) intervention study**: Eligible children with CP in the intervention group of the CURIE intervention study will be randomly assigned to receive PFT conditions so as to create the “both PD and PFT”, and “only PFT” conditions, as these individuals will receive PFT by default of the CURIE study. Likewise, participants in the CURIE control group will be randomly assigned to receiving, the “only PD condition”, and the “control” (neither PD nor PFT) condition.

**Randomization of participants from other organisations:** Since not all the CURIE intervention study participants may be eligible to participate (malnourished), other eligible caregivers and their children with CP sampled from elsewhere in the study site will be allocated to either the PFT or control groups of the CURIE study arms based on home village vicinity to make up the required sample for each of the four experimental conditions. Research assistants will be provided with the allocation of all participants after the baseline survey.

In order to avoid contamination, participants in the CURIE-intervention study have been allocated to clusters depending on the village where they reside. Neighboring villages were clustered together to reduce contamination between groups resulting in two geographically defined groups matched for age, sex and motor function. Given the similarity in study sites of the CURIE- study and this study, participants in our study will be allocated based on the already determined CURIE-intervention or control group village clusters as earlier described to avoid contamination. Participants in the control condition will be on waiting list to receive the PFT intervention later owing to the design of the wider CURIE study arrangement.

**Blinding:** Evaluators will be blinded on group allocations so that parent facilitators and caregivers do not report their subjective outcome measures differently. A group of independent outcome assessors (evaluators), not involved in the intervention, will administer the outcome measures after the interventions. These examiners will be blinded to group allocation to avoid reporting bias.

**Data management and analysis.**

Descriptive statistics will be performed by tabulation of the summary means of the individual condition levels and baseline social demographic characteristics of the children and their caregivers. Participants’ characteristics will be presented in frequency tables. A two-way analysis of variance (ANOVA) will be performed to test the independent main effects of PD and PFT, and the effect of interaction of both PD and PFT. Factor levels will be compared by a pairwise comparison of treatment means (standard error of a difference) or a fisher’s least significant difference (LSD) test. This analysis will be done in both intention to-treat and per-protocol assumptions. All analysis will be expressed in 95% two sided confidence intervals and a p-value of <0.05 will indicate statistical significance.

**Study flow chart:**

CP child -caregiver pairs

Normal

>-1SD WHZ, WAZ, HAZ

Malnourished

Screening

**Severe (exclusion)**

MUAC <11.5cm (Child),

<-3SD with Oedema

**Malnourished (inclusion)** N=124

<-2SD WHZ, HAZ, WAZ, MUAC

CURIE-No PFT control cluster

CURIE-PFT intervention cluster

-Caregiver counselling on immediate management of severe malnutrition

-Referral for management

Allocation and randomization

Follow-up

**Study Implementation plan:**

This study and preceding qualitative assessments will be implemented as follows: In the first month, a situation analysis (nutrition survey) will be conducted by trained data collectors or clinicians to identify well-nourished and malnourished children and adolescents with CP from selected organizations in the study area. In order to identify the unique practices of caregivers of well-nourished CP children identified from the nutrition survey, that set them apart from others within the same community, a qualitative survey will be conducted during the second month. From this qualitative survey, a Positive Deviance Inquiry (PDI) report that documents the identified successful practices and good foods will be generated. Based on the PDI report, a positive deviance (PD) program will be designed and evaluated against the Akwenda/CURIE caregiver training program (parent facilitator trainings -PFT) among malnourished CP children through a randomized clinical trial. The trial will be implemented for 3 months. Monthly Nutrition Education and Rehabilitation Sessions (NERS) for identified malnourished children and their caretakers under the PD program, and training sessions under the PFT program will be conducted concurrently. Monthly growth monitoring of all children in the study group will be done by an external team of assessors. In order to evaluate the main effect of the PD program and the PFT programs separately and in conjunction, data management, analysis and reporting will be done in the subsequent months.

**Table 1: A 2x2 table showing the allocations of the different experimental conditions in a factorial design.**

|  | PD-Yes | PD-No |
| --- | --- | --- |
| PFT-Yes | 31 (PFT-Yes, PD-Yes) | 31(PD-No, PFT-Yes) |
| PFT-No | 31 (PFT-No, PD-Yes) | 31 (PD-No, PFT-No) |
|  | 62 | 62 |

**7.0 Ethical Considerations**

Ethical approval for conducting the intervention study was obtained from Makerere University School of Public Health Higher degrees and Research Ethical Committee, and from the Uganda National Council of Science and Technology. Ethical approval for this study protocol will be sought from the same ethics institutions. The trial will be conducted according to the Declaration of Helsinki guidelines.

At the beginning of the surveys, all study participants aged 18 years and above will provide written informed consent. Child participants aged 8 years and above who are able to understand what the study is about will be taken through the assent process. For younger children, care givers will provide informed consent as participant proxies.

Every step will be taken to ensure confidentiality of data obtained. All collected data will be kept on a secure, encrypted laptop and accessed only by the study staff. Each survey will be given a unique identifier and no identifying information will be recorded. Risks and benefits of the study will be explained as outlined in the consent forms.

**COVID-19 risk management plan**

Infection control measures to minimize the risk of Covid-19 among the study team and participants will be implemented as per section 12 of the National guidelines for conduct of research during corona virus disease 2019 pandemic (July 2020) <https://www.uncst.go.ug/guidelines-and-forms/>. These measures include:

1. **Infection Control Measures at Research Institutions or Recruitment Entry**

**Points**

i). All persons presenting at the research institutions or recruitment site entry points should

wash their hands with soap and water or hand sanitizer that is provided at the entry

point.

ii). Screening of research teams and participants with an infrared temperature monitor at

research institutions or recruitment entry points.

iii). When screening/ examining research participants, the person in charge should have

appropriate Personal Protective Equipment (PPE) including a medical mask, gloves, gown

and eye protection (goggle or face shield) to protect themselves.

iv. Persons identified to have temperature≥37.5 C or respiratory/flu-like symptoms and/ or

history to an area with reported community transmission in the last 14 days before the

onset of symptoms (suspected/probable case), once identified shall be referred to district COVID-19 Taskforce teams.

**b). Physical Distancing**

i). A distance of a minimum of two (2) meters should be ensured during research procedures and activities to prevent person- to- person spread of the Corona Virus.

ii). No more than ten (10) people should converge in one place to undertake research

procedures. It is recommended that research teams ensure at least two (2) square meters

for each person. Research teams should avoid hand-shakes and hugging at all times.

**c). Handwashing and Disinfection**

Research teams and participants should consistently wash their hands with soap and water or use a hand sanitizer throughout the day when at the research institutions or recruitment sites until when they are ready to leave.

**d). Use of Personal Protective Equipment for Corona Virus Disease Prevention**

Personal Protective Equipment should be made available to all members of the research team that require it for their duties at the research sites. PPE for Coronavirus disease include gloves, medical/ surgical masks, goggles or face shield, gowns, aprons and medical grade gumboots. The use of PPE in the respective situations or risk categories will be tailored to the current guidelines of Ministry of Health.

**8.0 Study strengths and limitations**

To the best of our knowledge, this is the first experimental study to test the effect of the positive deviance strategy on the nutritional status of children and youth with CP in low-income countries (LICs). The other strength of this study is that the use of home visiting for data collection will allow for a naturalistic assessment of need and observation of the opportunities and barriers the caregiver may face with regards to feeding a child with CP. This will enable the immediate demonstration of advice ‘in situ’, and also help to build trust between the researchers and the families. However, this study had some limitations. Participant selection was primarily within the existing Akwenda/CURIE study two arm structure, and thus allocation of participants to study conditions was not completely random, which increased the potential for non-equivalent study groups and a high risk of bias. However, the difference in groups observed in some baseline factors was adjusted for during statistical analysis to determine the independent main effects of the PD and PFT interventions.

**References**

1. Kakooza-Mwesige A, Tumwine JK, Eliasson A-C, Namusoke HK, Forssberg H. Malnutrition is common in Ugandan children with cerebral palsy, particularly those over the age of five and those who had neonatal complications. *Acta paediatrica (Oslo, Norway : 1992)* 2015; **104**(12): 1259-68.

2. Black AP, D’Onise K, McDermott R, Vally H, O’Dea K. How effective are family-based and institutional nutrition interventions in improving children’s diet and health? A systematic review. *BMC Public Health* 2017; **17**(1): 818.

3. Sawyer W, Ordinioha B, Abuwa P. Nutrition intervention program and childhood malnutrition: a comparative study of two rural riverine communities in bayelsa state, Nigeria. *Ann Med Health Sci Res* 2013; **3**(3): 422-6.

4. Kim J, Lim H. Nutritional Management in Childhood Obesity. *J Obes Metab Syndr* 2019; **28**(4): 225-35.

5. Grant A, Njiru J, Okoth E, et al. Comparing performance of mothers using simplified mid-upper arm circumference (MUAC) classification devices with an improved MUAC insertion tape in Isiolo County, Kenya. *Archives of Public Health* 2018; **76**(1): 11.

6. D'Alimonte MR, Deshmukh D, Jayaraman A, Chanani S, Humphries DL. Using Positive Deviance to Understand the Uptake of Optimal Infant and Young Child Feeding Practices by Mothers in an Urban Slum of Mumbai. *Maternal and child health journal* 2016; **20**(6): 1133-42.

7. Bisits Bullen PA. The positive deviance/hearth approach to reducing child malnutrition: systematic review. *Tropical Medicine & International Health* 2011; **16**(11): 1354-66.

8. Bhattacharya S, Singh A. Using the concepts of positive deviance, diffusion of innovation and normal curve for planning family and community level health interventions. *J Family Med Prim Care* 2019; **8**(2): 336-41.

9. Kakooza-Mwesige A, Andrews C, Peterson S, Wabwire Mangen F, Eliasson AC, Forssberg H. Prevalence of cerebral palsy in Uganda: a population-based study. *The Lancet Global Health* 2017; **5**(12): e1275-e82.

10. Chantal Inamahoro JK, Fredrick Edward Makumbi, Gakenia Wamuyu-Maina, Henry Wamani. Nutritional Recovery Outcome among Moderately Malnourished Under-five Children in Communities Implementing Positive Deviance - Hearth or Community Health Workers’ Nutrition Promotion Approaches in Karusi and Kirundo Provinces, Burundi *Journal of science and sustainable development* 2017; **6**(1).

11. WHO. Child Growth Standards based on length/height, weight and age. *Acta paediatrica (Oslo, Norway : 1992) Supplement* 2006; **450**: 76-85.

12. Stevenson RD. Measurement of growth in children with developmental disabilities. *Developmental medicine and child neurology* 1996; **38**(9): 855-60.

13. WHO. WHO Anthro for personal computers, version 3.2.2, 2011. Software for assessing growth and development of the world’s children. . Geneva, Switzerland; 2010.

14. El Mouzan MI, Al Salloum AA, Alqurashi MM, Al Herbish AS, Al Omar A. The LMS and Z scale growth reference for Saudi school-age children and adolescents. *Saudi J Gastroenterol* 2016; **22**(4): 331-6.

15. Binns PJ, Dale NM, Banda T, Banda C, Shaba B, Myatt M. Safety and practicability of using mid-upper arm circumference as a discharge criterion in community based management of severe acute malnutrition in children aged 6 to 59 months programmes. *Arch Public Health* 2016; **74**: 24.

16. Marshall SK, Monárrez-Espino J, Eriksson A. Performance of mid-upper arm circumference to diagnose acute malnutrition in a cross-sectional community-based sample of children aged 6-24 months in Niger. *Nutr Res Pract* 2019; **13**(3): 247-55.

17. Alice M Tang MC, Kimberly Dong, Christine Wank, Zena Maalouf Manasseh. Determining a Global Mid-Upper Arm Circumferance Cutiff to assess Underweight in Adults (Men and Non-pregnant Women). Washington DC, 2017.

18. Health MO. Guidelines On Nutrition Survey Methodology In Uganda. In: Health MO, editor. Kampala Uganda; 2009. p. 42.

19. Onward M. Parents Training Parents in Rural South Africa.

20. Paulson A, Vargus-Adams J. Overview of Four Functional Classification Systems Commonly Used in Cerebral Palsy. *Children (Basel)* 2017; **4**(4): 30.
